# Recent advances in immunotherapy for gliomas: overcoming barriers and advancing precision strategies

**DOI:** 10.3389/fimmu.2025.1690464

**Published:** 2026-01-08

**Authors:** Abdullah Jabri, Abdulaziz Mhannayeh, Bader Taftafa, Mohamed Alsharif, Dania Sibai, Raghad Alsharif, Tasnim Abbad, Abdulrahman Elsalti, Zara Ahmed, Jahan Salma, Mohammed Imran Khan, Ahmed Yaqinuddin

**Affiliations:** 1 College of Medicine, Alfaisal University, Riyadh, Saudi Arabia; 2 College of Medicine, AlMaarefa University, Riyadh, Saudi Arabia; 3 International School of Medicine, Istanbul Medipol University, Istanbul, Türkiye; 4 Organ Transplant Center of Excellence, King Faisal Specialist Hospital and Research Center, Riyadh, Saudi Arabia; 5 King Faisal Specialist Hospital and Research Center, Jeddah, Saudi Arabia

**Keywords:** glioblastoma, glioma, immunotherapy, tumor microenvironment, immune checkpoint inhibitors

## Abstract

Gliomas, and particularly glioblastoma (GBM), remain among the most lethal primary brain tumors, with outcomes constrained by extensive intra tumor heterogeneity, a profoundly immunosuppressive tumor microenvironment (TME), and the restrictive nature of the blood–brain barrier (BBB). Although immunotherapies, including immune checkpoint inhibitors, chimeric antigen receptor (CAR) T and NK cells, and oncolytic virotherapy, have redefined treatment paradigms in other malignancies, their efficacy in gliomas has been modest, limited by low tumor mutational burden, antigenic plasticity, metabolic suppression, and therapy-associated immunosuppression. Recent advances in multi-antigen targeting, metabolic reprogramming, and innovative delivery strategies have enhanced preclinical efficacy, while the integration of emerging biomarkers such as ADAMTSL4, ACSS3, and radiomics-derived immune signatures offers opportunities for precision patient stratification. Converging developments in real-time molecular monitoring, spatial immunoprofiling, and rationally designed combination regimens hold the potential to recalibrate the glioma immune landscape, paving the way toward clinically impactful and durable immunotherapeutic responses.

## 1 Introduction

Glioblastoma multiforme (GBM) is the most common and aggressive brain tumor in adults, accounting for 49.1% of malignant CNS tumors (1). Despite the use of established modalities such as surgery, radiation, and chemotherapy, the prognosis remains poor, with a median survival of only 14 months and less than 5% of patients surviving beyond five years (2, 3). The major treatment approaches and their respective benefits and limitations are summarized in **Table 1**, which provides a concise overview of why conventional therapies have thus far failed to achieve durable outcomes.

**Table 1:** Standard-of-care glioma treatments and limitations.

Therapy	Mechanism	Clinical benefit	Key limitations	Reference(s)
Surgery	Maximal safe resection	Immediate tumor burden reduction	Incomplete resection due to infiltrative growth	(2, 3)
Radiotherapy	DNA damage induction	Local disease control	Neurotoxicity, radioresistance	(2, 3, 6)
Temozolomide	DNA alkylation	Survival benefit in MGMT-methylated tumors	Resistance in unmethylated tumors	(7, 8)
Bevacizumab	VEGF inhibition	PFS improvement, edema reduction	No overall survival benefit	(9, 10)

In this review, the term *glioma* is used broadly to refer to diffuse gliomas encompassing several distinct molecularly defined entities. These include IDH-wildtype glioblastoma (GBM), IDH-mutant astrocytoma, diffuse midline glioma (DMG)—typically characterized by H3K27 alterations—and low-grade gliomas. While these subtypes share glial origin and certain biological features, they differ significantly in molecular profile, prognosis, and therapeutic responsiveness. The term *GBM* herein specifically refers to IDH-wildtype glioblastoma, consistent with the 2021 WHO classification of CNS tumors.

The aggressiveness of GBM is largely driven by its complex molecular profile, which includes dysregulation of critical signaling pathways such as PI3K/AKT/mTOR, Wnt/NF-κB, and TGF-β. These alterations promote tumor invasion, therapeutic resistance, and recurrence (4). Another major obstacle is tumor heterogeneity, as diverse genetic and phenotypic subpopulations of GBM cells contribute to resistance against conventional therapies. Treatment efficacy is further limited by the blood–brain barrier (BBB) and the profoundly immunosuppressive tumor microenvironment (TME) (5, 6).

Temozolomide (TMZ), an oral alkylating agent, has remained a cornerstone of GBM therapy. It has demonstrated survival benefit in elderly patients with glioblastoma, anaplastic gliomas, and progressive ependymomas (7). In addition, TMZ has shown lower toxicity with comparable efficacy to radiation in high-risk low-grade gliomas and to platinum-based chemotherapy in pediatric high-grade gliomas (8). Bevacizumab, an anti-angiogenic agent targeting vascular endothelial growth factor (VEGF), has also been used for recurrent GBM (9). While randomized controlled trials have not demonstrated a significant overall survival benefit, bevacizumab has improved progression-free survival and provided symptomatic relief through reduction of tumor-associated edema. Its approval was based on evidence of durable responses in patients with progressive GBM following prior therapy. Despite these benefits, and as summarized in **Table 1**, none of the current therapeutic options has succeeded in producing sustained survival gains, underscoring the urgent need for new approaches (10).

These limitations of conventional therapies have shifted attention toward immunotherapy, which seeks to overcome the TME and mobilize the immune system against GBM (11). Immunotherapeutic strategies under investigation include immune checkpoint inhibitors (ICIs), chimeric antigen receptor (CAR) T and nature killer (NK) cells, cancer vaccines, and oncolytic virotherapy (12). However, their application in GBM has been hampered by challenges such as restricted BBB penetration, limited *in vivo* stability, and inadequate tumor-specific targeting (13). The growing interest in immunotherapy represents a direct response to the shortcomings outlined in **Table 1**, aiming to harness the precision and adaptability of the immune system. This paper highlights how recent advances in precision immunotherapy and novel biomarkers may help overcome these barriers, addressing a critical gap in the current literature. In this review, we discuss recently identified GBM biomarkers, such as ADAMTSL4 and ACSS3, and metabolic proteins associated with GBM immune suppression and poor prognosis and their potential to stratify patients for immunotherapy. We also highlight adaptive, personalized approaches (e.g. multi-antigen CAR-T constructs, radiomic immune signatures, liquid biopsy assays) that complement and extend the strategies in the earlier reviews. In summary, while covering the established therapies, our review uniquely emphasizes precision medicine innovations and patient-specific combination regimens.

## 2 Methods

This review was conducted as a narrative review with the aim of providing a comprehensive and timely synthesis of the immunological landscape of glioblastoma (GBM) and recent advances in immunotherapeutic strategies. We performed a structured literature search of PubMed and Web of Science through July 2025 using combinations of the following terms: glioblastoma, immunotherapy, immune checkpoint inhibitor, CAR T cells, CAR NK cells, oncolytic virus, cancer vaccine, dendritic-cell vaccine, biomarker, radiomics, ADAMTSL4, and ACSS3. Reference lists of key publications were also manually reviewed to identify additional relevant studies.

We included peer-reviewed clinical trials, preclinical studies with translational relevance, and high-impact reviews published in English. Priority was given to recently published phase I–III clinical trial reports, major updates from ongoing studies, and PubMed-indexed primary research articles. Duplicate reports and abstracts without full manuscripts were excluded.

As this is a narrative review, we did not perform a formal quality appraisal or quantitative meta-analysis. Instead, we aimed to critically summarize emerging themes, highlight pivotal positive and negative clinical trials, and identify gaps in translation. The scope emphasizes recent clinical trial results and novel biomarkers while acknowledging limitations of preclinical models.

## 3 The immunosuppressive glioma microenvironment

**Figure 1:**
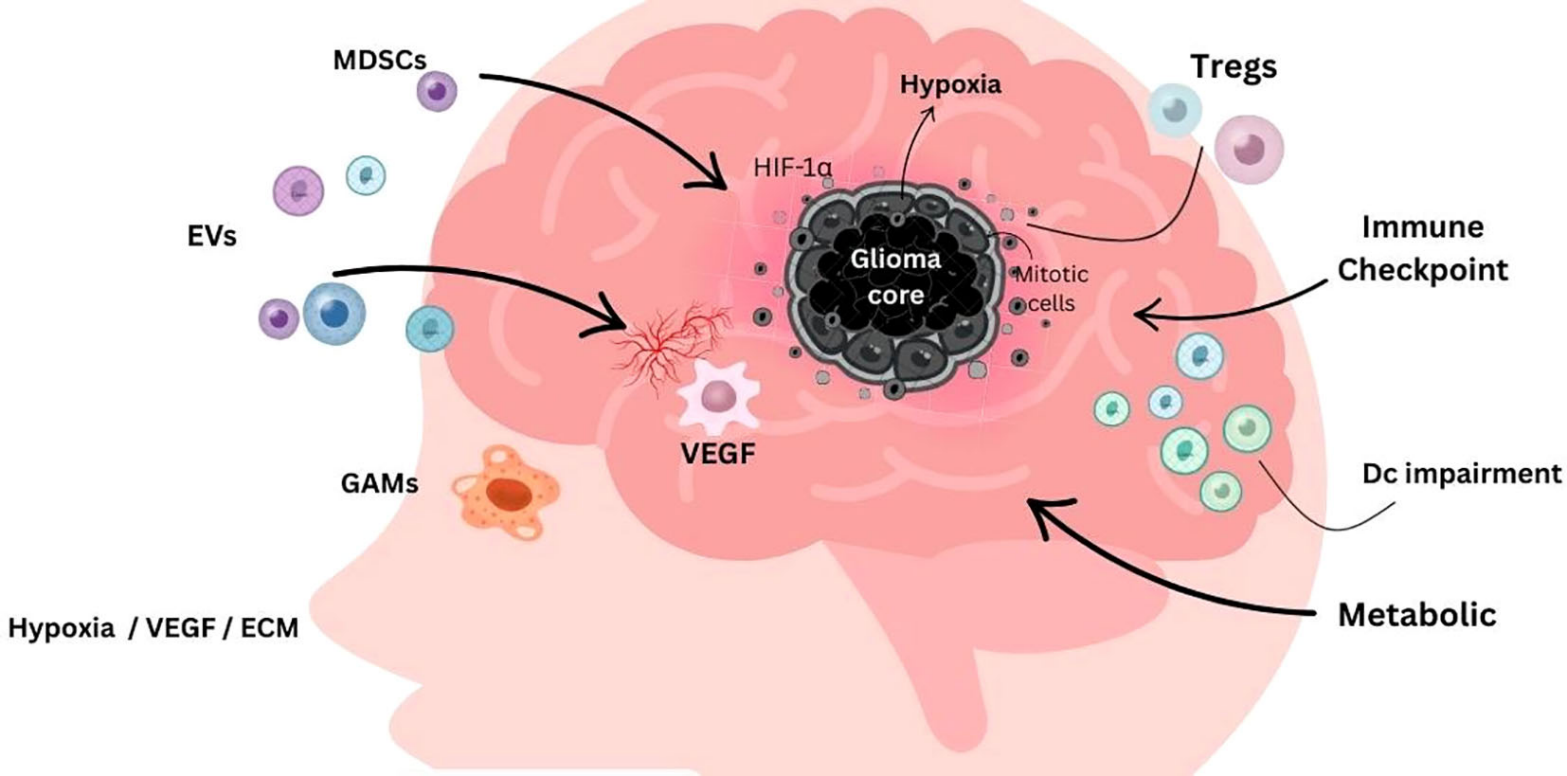
Immunosuppressive mechanisms within the glioma TME. Glioma cells employ a multi-layered strategy to suppress the immune response. Key players include MDSCs, M2-polarized GAMs, Tregs, and immunosuppressive EVs. These elements work in concert with metabolic stressors, checkpoint signaling, hypoxia, and the restrictive blood–brain barrier to block effective immune surveillance and therapy. Overcoming this environment requires combinatorial immunotherapy targeting both immune activation and TME remodeling.

The glioma TME, especially in high-grade gliomas like GBM, is highly immunosuppressive and poses a significant challenge to the effectiveness of immunotherapeutic treatments (14). This immunosuppressive milieu is created by a coordinated network of cellular, molecular, and anatomical pathways that allow tumor development while escaping immune-mediated elimination (15). Understanding the complexities of this milieu is critical for developing successful immunotherapies (16). **Figure 1** summarizes the mechanisms of immunosuppression in glioma TME.

### 3.1 Myeloid-derived suppressor cells

A central aspect of the glioma TME is the accumulation of immune cell populations that suppress the body’s natural antitumor responses (17). Among these, myeloid-derived suppressor cells (MDSCs) play a particularly important role (18). Even though they represent only a small fraction—typically around 4–8% of CD45^+^ immune cells in gliomas, their impact is disproportionately powerful (17). These cells inhibit T-cell activity through several mechanisms, including the expression of arginase-1 and inducible nitric oxide synthase (iNOS), the production of reactive oxygen species (ROS), and the release of anti-inflammatory cytokines such as interleukin-10 (IL-10) and transforming growth factor-beta (TGF-β) (19). In the hypoxic regions of the tumor, MDSCs can also differentiate into glioma-associated macrophages (GAMs), which contributes further to the dominance of the immunosuppressive M2-like macrophage phenotype in the glioma environment.

### 3.2 Glioma-associated macrophages

Gliomas are densely infiltrated by macrophages and microglia—together known as GAMs—which can account for as much as 30–50% of the tumor mass (15). Instead of triggering an immune attack against the tumor, these cells are often pushed into an M2-like immunosuppressive state. In this form, they show reduced ability to present antigens, secrete high levels of IL-10 and TGF-β, and actively support tumor progression by promoting blood vessel formation and remodeling the extracellular matrix (20, 21). Rather than helping the immune system eliminate cancer, M2-polarized GAMs suppress key immune cells such as cytotoxic T lymphocytes (CTLs) and dendritic cells (DCs). Glioma cells make matters worse by releasing factors like CSF-1, CCL2, and osteopontin, which further drive these macrophages into their tumor-supportive M2 state (22).

### 3.3 Regulatory T cells

In parallel, chemokine gradients (e.g., CCL22, CCL2) actively recruit regulatory T cells (Tregs) to the glioma TME, where they increase further under the effect of immunoregulatory enzymes such as indoleamine 2,3-dioxygenase (IDO) (23). Tregs inhibit immune responses by producing IL-10 and TGF-β, expressing inhibitory receptors such as CTLA-4, and inducing apoptosis of effector T cells via Fas ligand and perforin-dependent mechanisms (24). The net outcome is a strong suppression of anti-tumor immunity in the glioma environment.

### 3.4 Immune checkpoint pathways

Gliomas use immunological checkpoint pathways in addition to cellular components to suppress immune responses. Programed death ligand-1 (PD-L1), which binds to T cell PD-1 receptors and causes functional exhaustion, is highly expressed by glioma cells and invading immune cells (25). Multiple levels of suppression are produced by the upregulation of other immune checkpoint molecules in glioma-infiltrating lymphocytes, including TIM-3, LAG-3, VISTA, and cytotoxic T-lymphocyte-associated protein 4 (CTLA-4) (17).

### 3.5 Metabolic and cytokine-mediated suppression

Cytokine-mediated and metabolic processes exacerbate immunosuppression further. By absorbing vital resources like glucose and arginine, glioma cells actively alter the TME while depriving effector immune cells of the metabolic substrates necessary for activation and function (26). Gliomas concurrently emit immunomodulatory metabolites that promote Treg proliferation and suppress effector T-cell responses, including lactate, adenosine, and kynurenines (via IDO activity) (19, 27). Through the stability of hypoxia-inducible factors (HIFs), which stimulate the transcription of VEGF, PD-L1, and TGF-β, hypoxia, a characteristic of rapidly proliferating gliomas, intensifies these effects (28, 29).

### 3.6 Blood–brain barrier

The brain’s comparatively immune-privileged status, which is further strengthened by the BBB physiological and structural characteristics, adds to this intricacy. The BBB frequently stays intact in peripheral areas despite becoming somewhat disrupted in glioma cores, which restricts the infiltration of peripheral immune cells and the penetration of immunotherapeutics provided systemically (16). Furthermore, where immune infiltration would be most harmful to the tumor, glioma cells can control endothelial cells and pericytes to strengthen the integrity of the blood-brain barrier (14).

### 3.7 Extracellular vesicles

Growing evidence points to extracellular vesicles (EVs) as important players in immune suppression within gliomas. These tiny vesicles, released by glioma cells, are loaded with immunosuppressive proteins like PD-L1, galectin-9, and TGF-β, along with regulatory microRNAs and long non-coding RNAs. Once taken up by other immune cells, these EVs can reprogram them into more suppressive, less effective states (30). What makes EVs particularly concerning is that they don’t just act at the tumor site—they can circulate throughout the body, spreading immune suppression far beyond the brain.

All of this contributes to the creation of a highly immunosuppressive environment in gliomas—one shaped by a complex network of suppressive immune cells, immune checkpoint activation, metabolic and cytokine reprogramming, low oxygen levels, EVs, and the protective barrier of the BBB. Because of this layered defense, treating gliomas will likely require combination immunotherapies that not only boost the activity of immune cells, but also target and break down the many barriers that tumors use to shield themselves from the immune system (31–33).

### 3.8 Epigenetic reprogramming

GBM uses epigenetic mechanisms to remodel the TME and escape the immune system. Changes in DNA methylation, histone modifications, and RNA regulation reprogram both tumor and immune cells. This leads to accumulation of immunosuppressive MDSCs, tumor associated macrophages (TAMs), and Tregs. It also impairs antigen presentation and T cell activation (34, 35). Recent studies have shown that epigenetic immune remodeling in Glioblastoma stem cells generates two subtypes: a non-mesenchymal immune signature (Non-MESImm) and a mesenchymal-immune signature (MESImm) (36). The MESImm state shows upregulation of interferon stimulated genes and interferon regulatory factors (IRF1, IRF7, IRF8). It also has CCL2, which recruits immunosuppressive myeloid populations and reinforces immune evasion. Similarly, EZH2 (Enhancer of Zeste Homolog 2) is a histone methyltransferase that represses gene expression. In GBM, EZH2 is highly expressed and promotes tumor progression by silencing tumor suppressor genes and contributing to immune evasion. It does so partly by keeping microglia/macrophages in an immunosuppressive M2-like state (37). In addition, dysregulation of m6A RNA, an epigenetic mark on RNA that regulates gene expression after transcription, promotes tumor progression by modulating immune signaling and recruiting immunosuppressive TAMs (38). Together, these findings highlight how epigenetic reprogramming stabilizes an immunosuppressive tumor microenvironment in GBM and shapes tumor–immune interactions.

## 4 Therapeutic strategies in glioma immunotherapy

### 4.1 Immune checkpoint inhibitors in gliomas

#### 4.1.1 Intrinsic limitations of ICIs in glioma treatment

ICIs have not been effective in treatment of gliomas despite showing success in other cancers. Intrinsic tumor factors play a big role, with low TMB and consequent neoantigen paucity making most gliomas immunogenic except in hypermutated cases like bMMRD or POLE-mutant gliomas (39). This fundamental limitation was clinically validated in the CheckMate 143 and CheckMate 498 trials where nivolumab didn’t improve survival in recurrent or newly diagnosed glioblastoma patients (40). Other molecular barriers include IDH mutation associated epigenetic silencing of immune genes and low PD-L1 expression, and PTEN mutations and MAPK pathway aberrations that further decrease response (41, 42).

#### 4.1.2 Microenvironmental and therapeutic barriers

TME also contributes to ICI resistance in multiple ways. TBC1D1 overexpression causes profound T-cell dysfunction and blocks ICI response, while hypoxia driven PD-L1 upregulation creates an immunosuppressive niche (43, 44). Clinical management challenges exacerbate these biological barriers, especially the need for dexamethasone to control cerebral edema which systemically suppresses antitumor immunity (45). Current research is focused on overcoming these barriers through new combinations. Preclinical studies show that dual CTLA-4/PD-1 blockade can be synergistic, curing 75% of murine gliomas but clinical translation is challenging (46, 47). Engineered antibody approaches appear to be promising. For instance, Fc-enhanced anti-CTLA-4 agents like botensilimab can selectively deplete intratumoral Tregs through optimized FcγR binding (48). Metabolic interventions like lactate induced PD-L1 lactylation and nanodisc vaccines show potential to increase T-cell infiltration while reducing exhaustion markers (49, 50). Biomarker driven approaches are becoming more important as seen in the differential response of IDH-mutant vs hypermutated bMMRD gliomas to ICIs (42, 51). Ongoing early phase trials trials are evaluating CD47 blockade with magrolimab and IDO inhibition with indoximod to reprogram the immunosuppressive microenvironment and improve ICI response, however, efficacy signals remain preliminary (52, 53). While many promising metabolic interventions have been explored in glioma models, much of the preclinical evidence is derived from the murine GL261 glioma line. This model is notably more immunogenic than human GBM and differs in key microglial and immune marker expression, which may overestimate therapeutic efficacy. Translational caution is therefore warranted, and future studies in humanized models or patient-derived systems will be critical to validate whether findings such as lactylation inhibition or anti-CD47 synergy can be reliably extended to human glioblastoma. Large randomized trials such as CheckMate 143 and 498 likely underperformed due to design and biologic factors—including steroid exposure, lack of biomarker enrichment, and limited crossover—summarized with redesign principles in **Table 2**.

**Table 2:** Lessons learned from negative or neutral immunotherapy trials in GBM and redesign principles.

Trial (setting)	Key design limitations called out by field	Steroids (confounding)	Biomarker enrichment	Crossover	Endpoint/assessment issues	Lessons learned (why it failed to move OS)	Concrete redesign principles (what to do next)
CheckMate 143 (recurrent GBM; nivolumab vs bevacizumab)	Unselected population; bev arm reduces edema (symptom/QoL bias); BBB not addressed; late-line disease	High, variable; dexamethasone suppresses T-cell function and blunts ICI	None (no PD-L1/TMB/MMR selection)	No nivo crossover from bev arm	Pseudoprogression and nonstandardized iRANO use; OS primary	ICI given in steroid-exposed, unselected, late-line setting against an edema-control comparator → no OS gain	Steroid-sparing run-in; cap/record Dex dose; biomarker-enrich (bMMRD/POLE, high RIB, inflamed transcriptomes); neoadjuvant dosing pre-resection; allow protocolized crossover; imaging by iRANO with central review
CheckMate 498 (newly dx MGMT-unmethylated; RT+nivo vs RT+TMZ)	Removed TMZ entirely (possible loss of synergy/radiation-priming window); unselected; no BBB strategy	Frequent peri-RT steroids	MGMT only (no immune enrichment)	None	OS primary; no adaptive design	Immune-cold biology (IDH-WT, low TMB) + steroid exposure + lack of enrichment → nivo underperforms vs standard	Keep TMZ+RT backbone or add viral/metabolic priming; enrich for hypermutator/MMR-deficient; preplanned steroid minimization; peri-op neoadjuvant ICI window studies
CheckMate 548 (newly dx MGMT-methylated; RT+TMZ ± nivo)	No enrichment for inflamed tumors; heavy background therapy may mask ICI signal	Common	MGMT only	None	PFS/OS affected by pseudoprogression; limited immune monitoring	Signal diluted in unselected cohort with modest baseline immunogenicity	Prospectively select “hot” phenotypes (e.g., high RIB, TIL-high, PD-L1 myeloid signatures); embed serial immune monitoring; consider oncolytic-virus priming
ACT IV (rindopepimut; EGFRvIII vaccine)	Target antigen heterogeneity; antigen loss post-therapy; unselected beyond EGFRvIII	Variable	EGFRvIII only (no clonal stability check)	N/A	OS endpoint without durable antigen control	Antigen escape negated benefit	Multi-antigen vaccines; longitudinal antigen monitoring; combine with ICI/OV to curb escape
ICI monotherapy meta-experience (multiple phase II/III)	BBB penetration, myeloid-dominant PD-L1, low TMB; no microenvironmental reprogramming	Frequent	Rarely	Rare	Inconsistent iRANO use; imaging confounded by edema	Biology > drug: immune-cold, myeloid-suppressed GBM resists ICI alone	Combination first (ICI + OV/myeloid-targeted/metabolic); steroid-sparing, adaptive and window-of-opportunity designs; incorporate patient-level biomarker gating

### 4.2 CAR-T cell therapy for gliomas

#### 4.2.1 Antigen escape and immunosuppressive microenvironment

CAR-T cell therapy has many hurdles in glioma treatment. One major obstacle is antigen escape where gliomas downregulate target antigens such as EGFRvIII, IL13Rα2 and HER2 after treatment and CAR-T cells become ineffective (41, 54). This is worsened by the immunosuppressive TME where TBC1D1 positive macrophages and M2 polarized macrophages induce T cell exhaustion and actively suppress CAR-T cell activity (44). Metabolic barriers also hinder therapy as lactate accumulation upregulates immunosuppressive markers (CD39, CD73) and impairs CAR-T cell cytotoxicity (49). While the BBB has traditionally limited CAR-T cell infiltration, new delivery methods such as intratumoral or intraventricular administration show promise to overcome this hurdle (54, 55).

#### 4.2.2 Engineering solutions for enhanced efficacy

Clinical trials targeting IL13Rα2 and EGFRvIII have shown some responses but challenges remain with poor CAR-T cell persistence and systemic toxicities including cytokine release syndrome and neuroinflammation (40, 52). Researchers are working on new ways to improve CAR-T cell therapy in gliomas. Combinatorial approaches are showing promise with preclinical studies showing that radiation can improve blood brain barrier penetration and tumor immunogenicity (56). Metabolic interventions such as lactate inhibition through compounds like oxamate can restore CAR-T cell cytotoxicity by blocking immunosuppressive pathways (49). Next generation CAR-T designs are addressing antigen heterogeneity through bispecific and trispecific constructs targeting multiple antigens at once (e.g. HER2/IL13Rα2/EphA2) (46, 57). Genetic engineering approaches such as PD-1 dominant negative receptor (PD-1-DNR) modification can help CAR-T cells resist PD-L1 mediated suppression in the tumor microenvironment (54). For low grade gliomas, researchers are exploring mutant IDH1 targeted CAR-T cells as a potential therapy (51). Alternative approaches such as CAR-NK cells have advantages of being allogeneic and reduced risk of cytokine release syndrome (52, 54). Complementary neoantigen specific strategies such as sHDL nanodisc vaccines aim to generate robust CD8+ T cell responses against glioma targets (50). Recent advances in CAR T-cell therapy for gliomas highlight the potential of local delivery to overcome the blood–brain barrier (BBB) and improve therapeutic responses. A first-in-human study of intraventricular CARv3-TEAM-E cells, a next-generation CAR T product targeting EGFRvIII and secreting a T-cell–engaging anti-EGFR molecule, demonstrated rapid radiographic regressions in three patients with recurrent GBM after a single infusion, without dose-limiting toxicities or adverse events above grade 3. However, two of the three patients experienced transient responses, highlighting the challenge of durability in heterogeneous tumors (NCT05660369) (58).

Similarly, a phase I trial of GD2-targeting CAR T-cells for H3K27M-mutant diffuse midline gliomas (DMGs) employed a sequential intravenous (IV) and intracerebroventricular (ICV) delivery approach. The IV dose engaged the systemic immune response, while repeated ICV doses achieved high local CAR T-cell concentrations with minimal systemic toxicity. This approach led to major tumor volume reductions (52–100%) and significant neurological improvement, including one durable complete response ongoing for over 30 months, demonstrating the potential of local delivery to unlock durable anti-tumor activity in aggressive gliomas (Monje et al., 2024; PMID: 39537919). Together, these studies underscore the feasibility, safety, and therapeutic promise of CSF-based CAR T-cell delivery, while highlighting the need for strategies that enhance response durability in heterogeneous and aggressive brain tumors.

### 4.3 Oncolytic virotherapy approaches

#### 4.3.1 Mechanisms of action and clinical candidates

Oncolytic viruses (OVs) are an immunotherapeutic approach for gliomas that combines direct tumor cell killing with systemic antitumor immunity. The most advanced oncolytic virus candidates for glioma include G47Δ (Teserpaturev), an HSV-1-based virus which received conditional approval in Japan after a small, single-arm phase II trial (n=19) reporting a one-year survival rate of 92.3% in patients with recurrent glioblastoma, though without a randomized comparator. Similarly, PVSRIPO, a recombinant non-neurovirulent poliovirus chimera that demonstrated safety and durable responses in a subset of patients, but any survival benefit remains unconfirmed in randomized studies (59). These agents exploit tumor specific defects in antiviral defense mechanisms to selectively replicate and then release tumor associated antigens and damage associated molecular patterns that turn immunologically “cold” tumors into “hot” microenvironments by recruiting and activating dendritic cells and NK cells (60). Combining OVs with immune checkpoint inhibitors has particular therapeutic potential as viral infection induces IFN-γ mediated PD-L1 upregulation potentially making tumors more sensitive to anti-PD-1 therapy.

#### 4.3.2 Delivery challenges and next-generation vectors

Clinical implementation is challenging with limited blood brain barrier penetration - addressed through innovative delivery methods such as convection enhanced delivery or mesenchymal stem cell carriers - and pseudoprogression due to virus induced inflammatory responses. Research is focused on improving OV efficacy through genetic engineering and combination approaches. Next generation constructs armed with immunomodulatory transgenes such as IL-12 expressing HSV-1 (M032) show improved antitumor activity through reduced regulatory T cell infiltration and increased IFN-γ production and mesenchymal stem cell delivery improves tumor targeting in challenging anatomical locations such as diffuse intrinsic pontine glioma, though clinical validation is still ongoing (52, 59). Complementary metabolic interventions such as lactylation inhibition can counteract OV induced immunosuppressive cytokine secretion in the tumor microenvironment (49). Clinical results in pediatric populations are promising with OVs like G207 extending median overall survival to 12.2 months in high grade glioma compared to historical controls of 5.6 months. Persistent challenges include HLA class I dysregulation in gliomas which compromises neoantigen presentation and limits adaptive immune responses (42). Biomarker driven approaches such as CAN-3110 (an oncolytic herpes virus that only works in HSV-1 seropositive patients) highlight the importance of patient stratification (55). Low grade gliomas have unique immunological features such as “immune-quiet” microenvironments and IDH mutation driven immunosuppression which present additional challenges that require specialized therapeutic approaches.

A summary of key clinical and preclinical findings for these immunotherapeutic approaches is provided in **Table 3**. Detailed methodological and sample-size information are provided in **Supplementary Table S1**.

**Table 3:** Clinical evidence summary of immunotherapeutic strategies in gliomas.

Therapy type	Trial/agent	Design & phase	Primary endpoint	Biomarker/population	Level of evidence	Key findings/outcome	Reference
ICI	CheckMate 143 – Nivolumab vs Bevacizumab	Randomized Phase III	OS	Unselected recurrent GBM	Confirmatory (negative)	No OS benefit (mOS 9.8 vs 10.0 mo; HR 1.04)	(40)
ICI	CheckMate 498 – Nivolumab + RT vs TMZ + RT	Randomized Phase III	OS	Newly diagnosed MGMT-unmethylated GBM	Confirmatory (negative)	Inferior to TMZ + RT (mOS 13.4 vs 14.9 mo; HR 1.31)	(40)
Vaccine	DCVax-L (NCT00045968)	Externally controlled Phase III	OS, PFS	MGMT subgroup analysis	Moderate (non-randomized)	OS 19.3 vs 16.5 mo; limited by external controls	(98)
CAR-T	EGFRvIII-CAR-T	Single-arm Phase I	Radiographic response	EGFRvIII^+^ recurrent GBM	Exploratory	Safe; antigen loss → transient responses	(85)
CAR-T	IL13Rα2 CARv3-TEAM-E	Single-arm Phase I	Radiographic/neurologic response	IL13Rα2^+^ recurrent GBM	Exploratory	Rapid regressions (2 transient, 1 durable)	(58)
OV	G47Δ (Teserpaturev)	Single-arm Phase II	1-yr OS	Recurrent GBM	Preliminary (Approved Japan)	1-yr OS 84.2%; well tolerated	(59)
OV	PVSRIPO (poliovirus chimera)	Single-arm Phase I	OS	Recurrent GBM	Exploratory	Durable survival in subset	(54)
ICI + OV	DNX-2401 + Pembrolizumab	Open-label Phase I/II	Safety, OS	Recurrent GBM	Preliminary	Safe; signal of activity	(94)

## 5 Biomarkers and monitoring strategies for immunotherapy in gliomas

### 5.1 Limitations of established biomarkers: TMB, PD-L1, and TILs

Tumor Mutational Burden (TMB), PD-L1 expression, and TIL density are the most established biomarkers guiding the use of immune checkpoint inhibitors (ICIs) across various malignancies (61). These markers are routinely used in cancers like melanoma, non-small cell lung cancer, and urothelial carcinoma, where immunotherapy has shown consistent clinical benefit (62). In gliomas, however, these biomarkers are not clinically reliable despite being validated in other tumors, and their predictive value remains unproven in phase II/III glioma trials (63). Their utility in gliomas is limited and context-dependent due to the tumor’s low TMB, PD-L1 expression being largely myeloid-derived, and typically sparse TIL infiltration (64, 65).

In gliomas, PD-L1 is frequently expressed not by tumor cells but by tumor-infiltrating myeloid cells (TIMs), especially M2-like TAMs (66). This shifts the functional significance of PD-L1 from tumor cell-intrinsic to microenvironment-mediated adaptive resistance (67). While TILs-especially CD8+ cytotoxic T cells-are critical in many tumors, gliomas often exhibit sparse T cell infiltration (65). Even when present, CD8+ TILs are often rendered ineffective due to the immunosuppressive activity of PD-L1+ TAMs (66). Additionally, high TMB is present in only 3.5% of glioblastomas, further limiting the neoantigen-driven recruitment of effective antitumor T cells (64).

### 5.2 Emerging molecular biomarkers in glioma immunology

Recent studies have identified novel biomarkers that may overcome the limitations of traditional indicators like PD-L1, TMB, and TILs in glioblastoma. A summary of key emerging biomarkers is provided in **Table 4**. One such biomarker is ADAMTSL4, a secreted glycoprotein found to be enriched in IDH-wildtype and MGMT-unmethylated GBM. ADAMTSL4 expression correlates strongly with immune checkpoint molecules such as PD-1, PD-L1, and CTLA-4, as well as immune and stromal infiltration signatures. Despite not altering the proportion of specific immune cell types, high ADAMTSL4 expression was linked to lower tumor purity, a more complex immune microenvironment, and significantly poorer overall survival, indicating its potential as a prognostic and circulating biomarker for immune response in GBM (68–70). However, current evidence for ADAMTSL4 is limited to retrospective TCGA and other transcriptomic datasets, without prospective clinical validation or functional *in vivo* studies to confirm its predictive or prognostic utility (71).

**Table 4:** Emerging biomarkers for immunotherapy response in gliomas.

Biomarker	Mechanism	Association with immunotherapy response	Clinical/preclinical evidence	Reference(s)
ADAMTSL4	Secreted glycoprotein enriched in IDH-wt/MGMT-unmethylated GBM; correlates with immune checkpoints (PD-1, PD-L1, CTLA-4) and stromal infiltration.	High expression linked to immunosuppressive microenvironment, lower tumor purity, and poorer OS. Potential circulating biomarker.	Preclinical: Correlates with immune infiltration but not specific cell types. Prognostic in TCGA data.	(61–63)
ACSS3	Fatty acid metabolism regulator; promotes M2 macrophage/Treg infiltration and immune checkpoint (PD-1, CTLA-4, LAG3) expression.	High ACSS3 → immunosuppressive TIME, poor OS. May predict resistance to ICIs.	Preclinical: TCGA/CGGA cohort analysis; linked to EMT and immune evasion pathways.	(64)
CD81 (m6A-associated)	Member of a 10-gene immune signature; promotes GBM proliferation, migration, and stemness.	High-risk signature (incl. CD81) → activated IFN-γ, STAT3, EMT pathways. Potential target for combo therapy.	Preclinical: Validated *in vitro* (proliferation, apoptosis assays). Prognostic in GBM cohorts.	(65)
Radiomics Immunological Biomarker (RIB)	MRI-based quantification of M2-like TAM infiltration.	Stratifies tumors into “hot,” “cold,” or “super-cold” phenotypes. “Super-cold” may benefit from DC vaccines.	Clinical: Predicted survival benefit in DC vaccine trials (e.g., NCT02010606).	(71)
Exosomal miR-21	miRNA carried by tumor-derived exosomes; modulates immune evasion.	Differentiates pseudoprogression from true progression post-immunotherapy.	Clinical: Detected in serum; correlates with recurrence.	(72, 73)
Annexin V+ Microvesicles/ctDNA	Circulating tumor-derived material reflecting molecular heterogeneity.	Early detection of recurrence; dynamic monitoring of clonal evolution during therapy.	Clinical: ctDNA shows concordance with tissue biopsies.	(74)

Another novel biomarker, ACSS3, has been identified as a prognostic and immune-related marker in glioma. High ACSS3 expression correlates with poor overall survival and increased infiltration of immunosuppressive cells, including M2 macrophages and regulatory T cells. It is also strongly associated with immune checkpoints such as PD-1, CTLA-4, and LAG3. Enrichment analysis links ACSS3 to immune regulation, fatty acid metabolism, and epithelial–mesenchymal transition, suggesting its role in promoting an immunosuppressive microenvironment and predicting responsiveness to immunotherapy (72). Similar to ADAMTSL4, ACSS3 findings are based on descriptive bioinformatic correlations rather than mechanistic studies, and no wet-lab or prospective clinical evidence currently supports its application in patient stratification (71).

**Figure 2:**
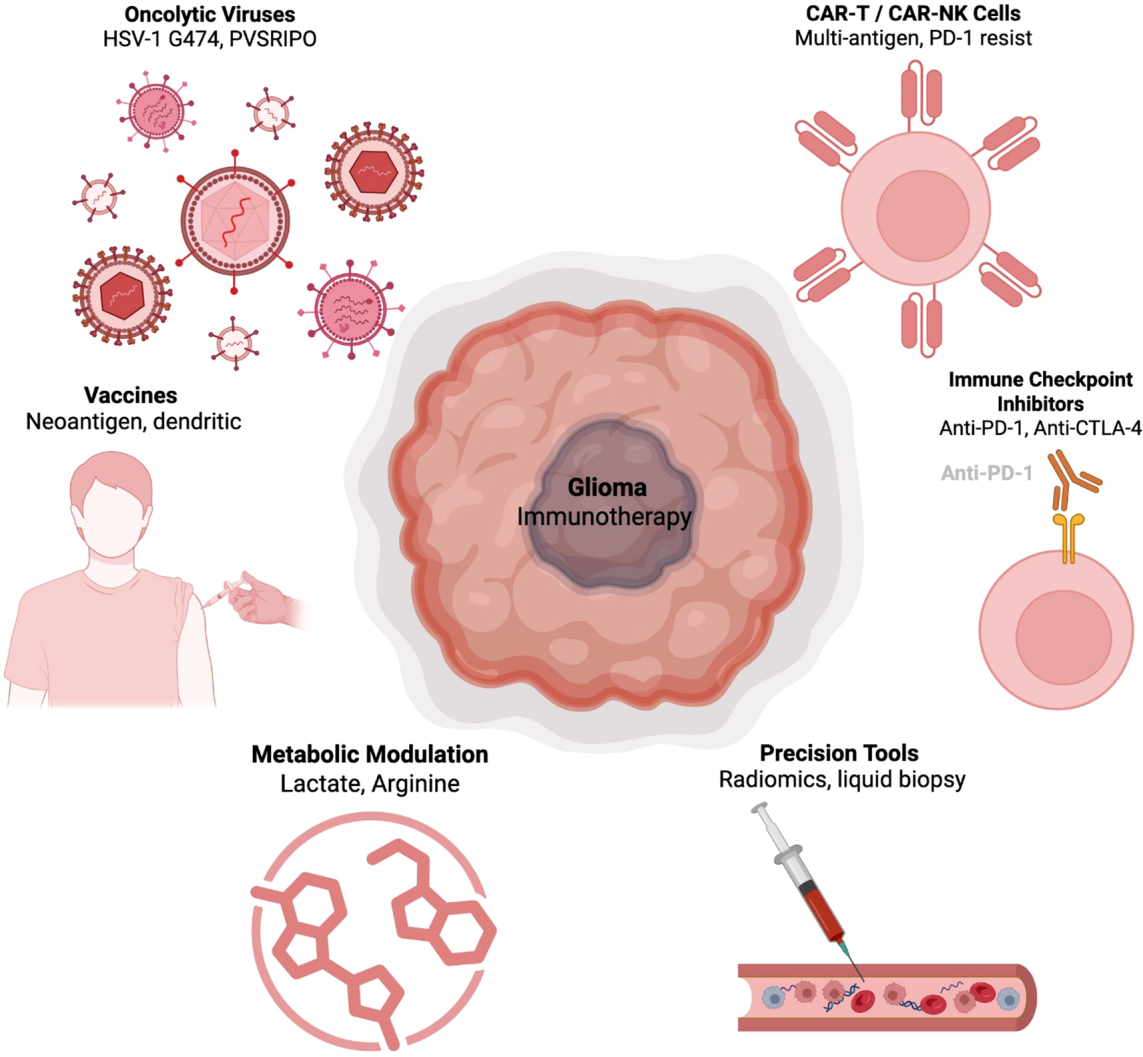
Emerging immunotherapeutic strategies for glioblastoma. This schematic highlights the diverse approaches currently under investigation to enhance glioblastoma immunotherapy. Strategies include oncolytic viruses (e.g., HSV-1 G474, PVSRIPO) that selectively infect and lyse tumor cells, vaccines (neoantigen and dendritic-based) designed to stimulate tumor-specific immunity, and engineered CAR-T or CAR-NK cells with multi-antigen targeting and PD-1 resistance. Immune checkpoint inhibitors such as anti–PD-1 and anti–CTLA-4 antibodies aim to restore T-cell activity, while metabolic modulation (e.g., lactate and arginine targeting) seeks to reprogram the tumor microenvironment. Precision tools such as radiomics and liquid biopsy further enable patient stratification and treatment monitoring, supporting personalized immunotherapy.

Lou et al. identified a prognostic model for glioblastoma based on ten m6A-associated immune genes. Patients were stratified into high- and low-risk groups with significantly different overall survival. Functional enrichment analysis of the high-risk group revealed activation of immune-related pathways, including interferon-γ response, IL6–JAK–STAT3 signaling, and epithelial–mesenchymal transition. Among the ten genes, CD81 was selected for experimental validation. *In vitro* assays demonstrated that CD81 promotes glioblastoma cell proliferation, migration, and stemness while inhibiting apoptosis, supporting its potential as a novel immune-related biomarker (73). Nonetheless, this validation remains limited to cell culture experiments, and further *in vivo* and clinical studies are required to establish CD81 as a reliable biomarker (71). These emerging biomarkers aim to better select patients for the advanced immunotherapeutic strategies summarized in **Figure 2**.

In summary, these emerging biomarkers provide a valuable framework for identifying immunologically distinct GBM subgroups, but their current utility is largely exploratory. Prospective clinical validation, functional *in vivo* studies, and integration with existing immunotherapeutic strategies remain critical before these markers can be applied in routine patient care.

### 5.3 Non-invasive tools for immune monitoring: radiomics and liquid biopsy

Radiomics offers a non-invasive means to characterize the tumor immune microenvironment (TIME) and monitor immunotherapy response in gliomas (71). By extracting quantitative imaging features, radiomics enables longitudinal assessment of immune activity and spatial heterogeneity, overcoming the limitations of tissue (74, 75).

Recent models have shown clinical relevance. A radiomic signature correlated with immune-related lncRNA expression and infiltration by CD8+ T cells, M2 macrophages, and resting memory CD4+ T cells, alongside immune checkpoint expression (PD-L1, CTLA-4), highlighting its potential for immunotherapy stratification in GBM (76). In another study, MRI-based texture features from early treatment scans predicted survival in glioblastoma patients receiving durvalumab, suggesting radiomics may reflect early immune-related changes (77).

A notable advancement is the Radiomics Immunological Biomarker (RIB), which quantified M2-like TAM infiltration and stratified gliomas into “hot,” “cold,” and “super-cold” immune phenotypes. Importantly, it predicted survival benefit from dendritic cell vaccine immunotherapy in patients with “super-cold” tumors (78).

Liquid biopsy (LB) offers a minimally invasive means to assess glioma molecular profiles, especially in cases where tissue access is limited. Among the most promising biomarkers are exosomal miRNAs, such as miR-21, which can differentiate between pseudoprogression and true recurrence (79, 80).

**Figure 3:**
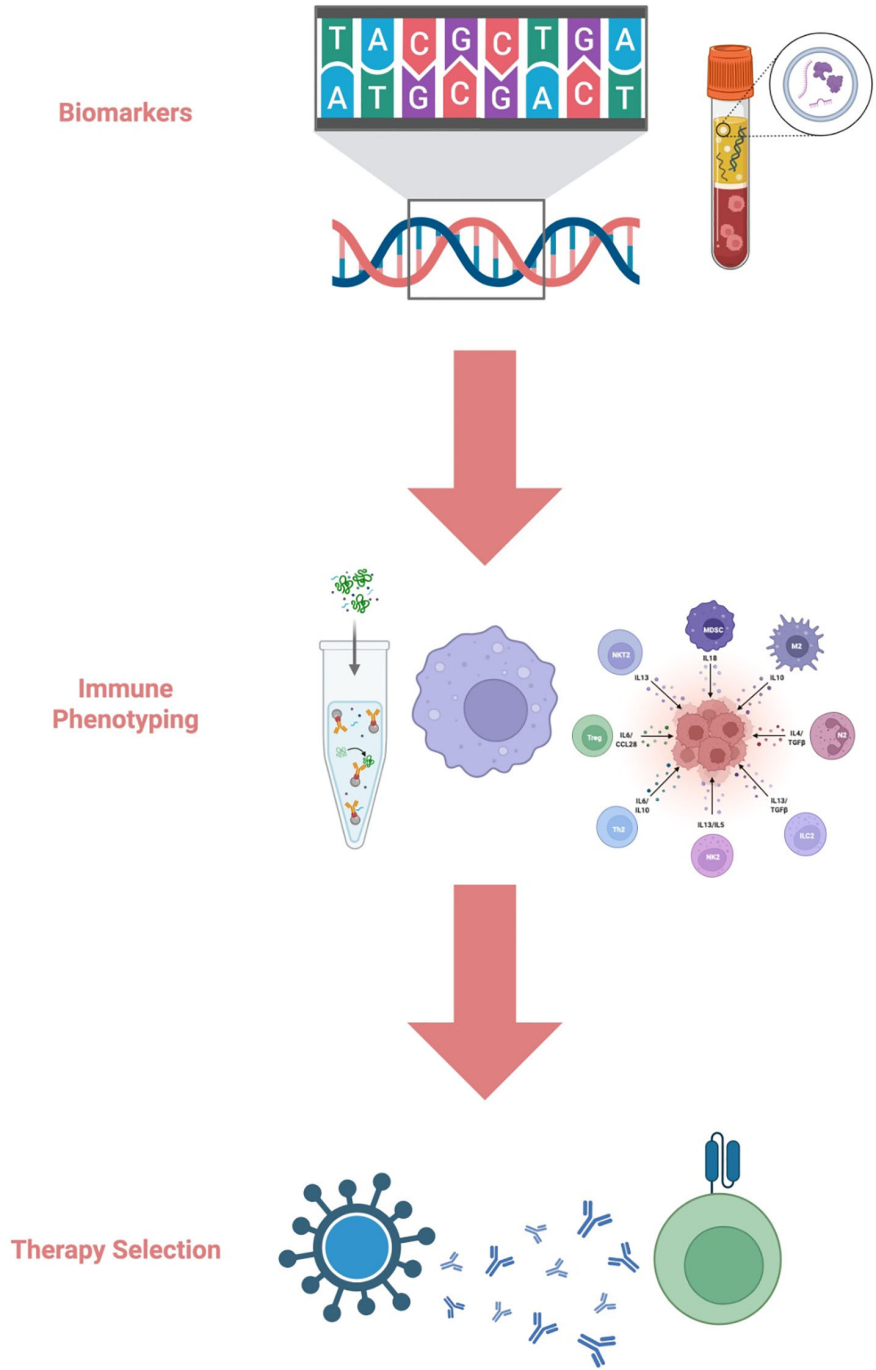
Precision-guided Immunotherapy Workflow in Glioblastoma. This schematic illustrates the process of tailoring immunotherapy for glioblastoma patients. Biomarker assessment, including genomic and blood-based analyses, serves as the initial step in identifying tumor-specific molecular alterations. Immune phenotyping further characterizes the tumor microenvironment and circulating immune populations to determine the immune landscape and functional deficits. Together, these approaches inform personalized therapy selection, enabling the rational choice of strategies such as immune checkpoint inhibitors, CAR-T/NK cell therapy, cancer vaccines, or antibody-based treatments.

Annexin V-positive microvesicles and circulating tumor DNA (ctDNA) have also shown potential in early recurrence detection and molecular monitoring (81). Further optimization of isolation techniques and BBB penetration may enhance their diagnostic performance. Together, radiomics and liquid biopsy form the core of a non-invasive monitoring paradigm, as depicted in **Figure 3**.

**Figure 4:**
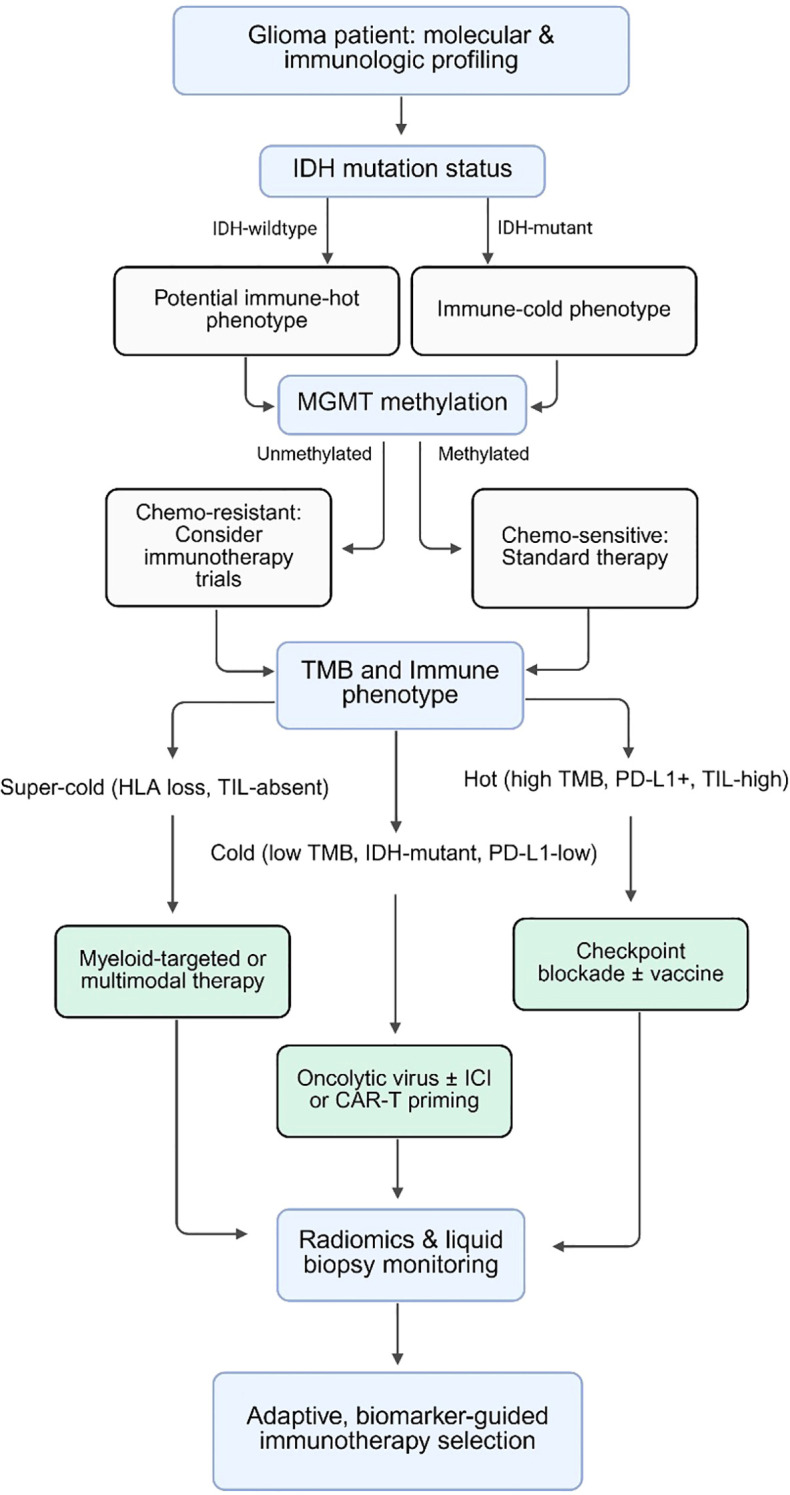
Precision immunotherapy decision framework for glioma.The flowchart summarizes how molecular and immune biomarkers can guide immunotherapy selection. IDH and MGMT define tumor subtype and chemoresponsiveness. Integration of TMB and immune phenotype (hot, cold, super-cold) informs therapy choice, while radiomic and liquid-biopsy markers support adaptive, biomarker-guided immunotherapy. IDH, isocitrate dehydrogenase; MGMT, O6-methylguanine-DNA methyltransferase; TMB, tumor mutational burden; PD-L1, programmed death-ligand 1; TIL, tumor-infiltrating lymphocytes; HLA, human leukocyte antigen; ICI, immune checkpoint inhibitor; CAR-T, chimeric antigen receptor T-cell.

To illustrate the integration of these molecular and imaging biomarkers into a clinical framework, **Figure 4** presents a conceptual decision flowchart summarizing how IDH mutation, MGMT methylation, TMB, and immune phenotype (hot, cold, super-cold) can be sequentially integrated to guide immunotherapy selection. Radiomic and liquid-biopsy signatures are incorporated as non-invasive layers for adaptive monitoring.

## 6 Challenges and future directions

Treating GBM remains one of the greatest challenges in modern oncology, primarily due to its profound intratumoral heterogeneity. Within a single tumor, distinct cellular subpopulations exhibit variable metabolic phenotypes—including glycolysis, glutaminolysis, and fatty acid oxidation—each creating localized immunosuppressive niches that impair immune infiltration and therapeutic efficacy (82, 83). This diversity disrupts uniform therapeutic targeting and contributes significantly to immune evasion.

### 6.1 Antigen escape and tumor evolution

One of the most well-documented consequences of this heterogeneity is antigen escape. Studies utilizing single-cell RNA sequencing and spatial transcriptomics have shown that under the selective pressure of immunotherapies such as CAR T cells or monoclonal antibodies targeting EGFRvIII and IL13Rα2, tumor subclones can downregulate these target antigens. This enables resistant cell populations to expand, ultimately driving disease recurrence (84, 85).

### 6.2 Modeling tumor–immune interactions

Progress in treatment development has been hindered in part by limitations of conventional mouse models like GL261, which fail to replicate human immune responses and antigenic landscapes (86). However, humanized patient-derived xenograft (PDX) models, particularly those using bone-liver-thymus (BLT) mice, have emerged as promising platforms for co-engrafting human tumors and immune cells, thereby enabling the study of clinically relevant immune-tumor interactions (83).

In parallel, patient-derived organoid–immune co-culture systems preserve the original tumor architecture and antigen heterogeneity while allowing direct evaluation of therapeutic responses ex vivo. These models hold particular promise for preclinical screening of immune-based therapies on an individualized basis (83, 86).

### 6.3 Metabolic barriers and immune suppression

Beyond antigenic variability, GBM exhibits metabolic plasticity that contributes to immunosuppression. Glioma cells modify their use of key nutrients, including glucose, lipids, and amino acids such as tryptophan and glutamine, leading to nutrient deprivation, lactic acidosis, and impaired T-cell function in the tumor microenvironment (87, 88). These mechanisms suppress immune responses and foster treatment resistance. Metabolic interference with T-cell activation and proliferation has been well documented, particularly in the context of altered glycolytic flux and arginine depletion (26). Caveat on IDO1 inhibition. Despite compelling preclinical data implicating the tryptophan–IDO1–kynurenine axis in T-cell dysfunction, clinical translation has been disappointing to date. The phase 3 ECHO-301/KEYNOTE-252 trial of epacadostat plus pembrolizumab in melanoma did not improve outcomes versus pembrolizumab alone, leading to broad reappraisal of this strategy across solid tumors (89). To our knowledge, no epacadostat-based regimen has produced a positive efficacy signal in GBM, and ongoing development in this disease remains investigational. Accordingly, in GBM the IDO1 pathway should be framed as a hypothesis-generating target, not a clinically validated approach (**Box 1**).

Box 1 **Lessons from unsuccessful or limited-translation immunotherapeutic strategies**

Despite encouraging preclinical findings, several immunotherapy approaches in glioma have not translated into clinical benefit:

• IDO1 inhibition: The phase III ECHO-301/KEYNOTE-252 trial of epacadostat plus pembrolizumab in melanoma was negative, and no IDO1-targeting regimen has produced a positive efficacy signal in glioblastoma.

• EGFRvIII vaccination (rindopepimut): The phase III ACT IV trial failed to improve overall survival in newly diagnosed GBM, underscoring the challenge of antigen heterogeneity.

• Checkpoint-inhibitor monotherapy: Large randomized trials (CheckMate 143, 498, 548) showed no overall-survival benefit over standard therapy.

These experiences illustrate the gap between preclinical promise and clinical outcome and highlight the importance of biomarker-guided, combination-based trial designs in future glioma immunotherapy research.

### 6.4 Hypoxia and checkpoint pathway crosstalk

Hypoxic conditions, a hallmark of rapidly proliferating gliomas, further enhance immunosuppressive signaling through the stabilization of hypoxia-inducible factors (HIFs), which drive transcription of PD-L1, VEGF, and TGF-β (28). In this setting, glioma cells also upregulate immune checkpoint molecules such as PD-L1 in response to HIF-1α, leading to functional exhaustion of infiltrating T cells (90). Recent studies have further emphasized the molecular crosstalk between hypoxia pathways and PD-1/PD-L1 signaling, revealing complex feedback loops that sustain immune evasion in gliomas (91).

### 6.5 Why ICIs underperform

Although ICIs have revolutionized cancer immunotherapy, they have shown limited efficacy in gliomas. This is largely attributed to the immunosuppressive tumor microenvironment and cellular heterogeneity that blunts ICI responses. Systematic reviews have confirmed the lack of significant survival benefits in unselected GBM populations, highlighting the need for combination strategies that address both intrinsic and extrinsic resistance mechanisms (92).

### 6.6 Toward adaptive and personalized immunotherapies

Key strategies under investigation to overcome these challenges are summarized in **Table 5**. Recent advances in single-cell and spatial transcriptomics have made it possible to monitor tumor evolution in real time, enabling the design of adaptive immunotherapies responsive to changing antigenic and metabolic landscapes (82). Promising strategies include the development of multi-antigen CAR T cells that target several tumor-associated antigens simultaneously, reducing the risk of antigen escape (84, 85). Spatial surveillance technologies allow early detection of antigen-loss variants and enable treatment recalibration. Moreover, therapies that combine immune checkpoint inhibition with metabolic reprogramming agents offer potential for remodeling the tumor microenvironment to facilitate immune cell infiltration and function (87, 93). Targeting distinct metabolic dependencies within the tumor—such as glutamine metabolism in nutrient-rich zones and glycolysis in hypoxic regions—further exemplifies the promise of personalized metabolic therapy (88). To emphasize that gliomas encompass immunologically distinct molecular entities, **Table 6** summarizes the immune‐landscape characteristics, dominant biomarkers, and immunotherapeutic strategies specific to each major subtype—IDH-wildtype glioblastoma, IDH-mutant astrocytoma, diffuse midline glioma (DMG), and low-grade gliomas. This table clarifies which biomarkers and treatment approaches are most relevant to each subtype.

**Table 5:** Future and adaptive immunotherapy strategies for gliomas.

Strategy	Approach	Mechanism/rationale	Examples/agents	Current status	Reference(s)
Multi-Antigen CAR-T Designs	Targeting multiple tumor-associated antigens (TAAs) simultaneously.	Reduces antigen escape by covering heterogeneous subclones (e.g., EGFRvIII, IL13Rα2, HER2).	Bivalent CAR-T (EGFR/IL13Rα2), Trivalent CAR-T (EGFR/HER2/EPHA2).	Phase I Trials (NCT05168423, NCT05660369).	(77, 78)
Metabolic Modulation	Inhibiting immunosuppressive metabolites (lactate, kynurenine) or nutrient scavenging.	Reverses T-cell exhaustion by alleviating hypoxia/acidity; blocks arginase/IDO-mediated suppression.	LDHA inhibitors (e.g., stiripentol), IDO inhibitors (epacadostat), arginase blockers (CB-1158).	Preclinical/early-phase trials (NCT04049669). Phase 3 ECHO-301/KEYNOTE-252 melanoma trial was negative, and epacadostat-containing regimens in GBM have not produced efficacy signals (e.g., NCT03707457). These agents should be considered investigational and non-promising pending new data	(80, 81, 89)
Hypoxia-Targeted Combos	Combining ICIs with HIF-1α inhibitors or anti-angiogenics.	Disrupts HIF-1α→PD-L1/VEGF axis; normalizes vasculature for immune infiltration.	Pembrolizumab + bevacizumab; durvalumab + HIF-1α inhibitor (PT2977).	Phase II trials (NCT03890952).	(83, 84)
Organoid-Guided Therapy	Using patient-derived organoids to test drug combinations ex vivo.	Preserves tumor heterogeneity/immune context; predicts personalized responses.	Organoid + autologous T-cell co-cultures; high-throughput drug screening.	Research/translational studies.	(76, 79)
Spatial Immunoengineering	Local delivery of immunotherapies (e.g., ultrasound-enhanced BBB opening).	Overcomes poor CNS drug penetration; targets regional immune niches.	Focused ultrasound (FUS) + anti-PD-1; intracranial CAR-T infusion.	Phase I/II trials (NCT03744026, NCT04225039).	(86)

**Table 6:** Subtype-specific immune landscapes and biomarker relevance in gliomas.

Glioma subtype	Key immune landscape features	Relevant biomarkers	Promising immunotherapeutic strategies	Notes
IDH-wildtype GBM	Highly immunosuppressive TME; abundant TAMs, PD-L1^+^ myeloid cells, low TILs, high HLA dysregulation	TMB (high in bMMRD/POLE cases), ADAMTSL4↑, ACSS3↑	Checkpoint blockade ± oncolytic virus; DC vaccine for MGMT-methylated tumors	Represents ~90% of high-grade gliomas
IDH-mutant astrocytoma	“Immune-cold”; low TMB, 2-HG-mediated T-cell suppression, minimal PD-L1	IDH1 mutation, low ACSS3 expression	Mutant IDH1 vaccines; metabolic modulation; CAR-T targeting IDH1	Usually low-grade or secondary GBM
Diffuse Midline Glioma (H3K27M)	Pediatric predominance; immune-excluded microenvironment; H3K27M mutation modifies antigen presentation	H3K27M, GD2	GD2-CAR-T ± intracerebroventricular delivery; oncolytic virus therapy	Clinical trials show durable responses in subset
Low-Grade Gliomas (grade 2–3)	Immune-quiet TME; rare TILs; slow growth	IDH1/2, MGMT, TERT	Mutant IDH1 vaccine trials; immune priming combos	Translational models under development

Ongoing clinical evaluation increasingly emphasizes rational combinations that reflect the outlined biology. Intratumoral DNX-2401 administered with pembrolizumab in recurrent glioblastoma demonstrated acceptable safety and signals of activity in the phase 1/2 CAPTIVE/KEYNOTE-192 trial (94). Neoadjuvant PD-1 blockade prior to resection increased interferon-γ–associated programs, enhanced intratumoral T-cell responses, and was associated with improved overall survival in a randomized design (95). By contrast, large, randomized studies of PD-1 monotherapy have been negative: nivolumab did not improve overall survival versus bevacizumab in recurrent disease and nivolumab with radiotherapy did not surpass temozolomide-radiotherapy in newly diagnosed, MGMT-unmethylated glioblastoma (96, 97). Vaccine strategies are also advancing; a pivotal development was the phase 3 trial of DCVax-L, a patient-specific dendritic-cell vaccine. This prospective, externally controlled, nonrandomized study demonstrated prolonged overall survival in both newly diagnosed and recurrent GBM. Among patients with newly diagnosed GBM, median OS was 19.3 months from randomization with DCVax-L (22.4 months from surgery) compared with 16.5 months in matched external controls (HR 0.80; P = .002). In recurrent GBM, median OS was 13.2 versus 7.8 months, respectively (HR 0.58; P <.001). Subgroup analyses suggested greater benefit in MGMT-methylated tumors, and the safety profile was favorable (98). While the externally controlled design precludes definitive causal inference, these findings provide the strongest clinical evidence to date for dendritic-cell vaccination in GBM and support ongoing efforts to integrate vaccines with checkpoint inhibitors or metabolic interventions to counteract antigen loss and immune suppression. Together, these data support biomarker-guided, multi-mechanistic regimens that combine multi-antigen targeting, checkpoint blockade, and metabolic or microenvironmental modulation in prospective trials.

By integrating organoid-based platforms, humanized *in vivo* models, and spatially guided immunoengineering, the field is advancing toward highly individualized, durable treatments for GBM that can adapt in parallel with the evolving biology of the tumor.

## 7 Conclusion

Despite years of incremental progress, gliomas – especially GBM – remain the ultimate test of modern oncology and can withstand even the most promising immunotherapies. Their resistance is multi-faceted: antigenic heterogeneity to escape immune recognition, a TME dominated by myeloid derived suppressor cells, M2 polarized macrophages and regulatory T cells and deep metabolic and hypoxic constraints that blunt effector cell function. Recent trials have shown that single agent interventions are not enough; the future of glioma immunotherapy will depend on integrative approaches that combine immune activation with disruption of microenvironmental resistance. Advances in localized and multi-antigen CAR-T, stem cell mediated oncolytic virus delivery and metabolic modulation are converging with precision tools like radiomics, liquid biopsy and spatial transcriptomics to enable adaptive, patient specific regimens. By combining these innovations within a biomarker guided framework we can move immunotherapy for gliomas from an exciting concept to a clinical reality.

In the end, accurate and flexible techniques will be the key to real breakthrough in glioma immunotherapy. Treatments can adapt to the tumor’s microenvironment and evolution by combining novel therapeutic designs with biomarkers, radiomics, liquid biopsies, and real-time monitoring. With the help of this adaptive precision framework, patients may finally experience long-lasting and significant clinical benefits.
