# Supplementary material for: Recent advances in immunotherapy for gliomas: overcoming barriers and advancing precision strategies
Source: Front Immunol. 2026 Jan 8;16:1690464. doi: 10.3389/fimmu.2025.1690464 (PMC12823976; doi:10.3389/fimmu.2025.1690464)
Supplement: Supplementary file 1 [file Table1.docx]

**10 Supplementary Material**

Table S1: Expanded Clinical Evidence Summary

| **Therapy Type** | **Trial / Agent** | **Design** | **Patient Population** | **Sample Size (n)** | **Primary Endpoint** | **Biomarker Stratification** | **Phase / Level of Evidence** | **Key Findings / Outcome** | **Status / Reference** |
| --- | --- | --- | --- | --- | --- | --- | --- | --- | --- |
| ICI | CheckMate 143 – Nivolumab vs Bevacizumab | Randomized, controlled, open-label | Recurrent GBM | 369 (184 Nivo, 185 Bev) | OS | None (unselected) | Phase III – Confirmatory (negative) | No OS benefit (mOS 9.8 vs 10.0 mo; HR 1.04, P = 0.76) | Completed [40] |
| ICI | CheckMate 498 – Nivolumab + RT vs TMZ + RT | Randomized, controlled | Newly diagnosed MGMT-unmethylated GBM | 560 (280 per arm) | OS | MGMT status | Phase III – Confirmatory (negative) | Inferior to TMZ+RT (mOS 13.4 vs 14.9 mo; HR 1.31, P = 0.0037) | Completed [40] |
| Vaccine | DCVax-L (NCT00045968) | Externally controlled, non-randomized | Newly diagnosed & recurrent GBM | 331 vaccine + 1366 controls | OS (primary), PFS (secondary) | MGMT methylation subgroup | Phase III – Non-randomized (Moderate evidence) | mOS 19.3 vs 16.5 mo (HR 0.80; P = 0.002); favorable safety | Completed [98] |
| CAR-T | EGFRvIII-CAR-T (O’Rourke et al., 2017) | Single-arm, open-label | Recurrent EGFRvIII⁺ GBM | 10 | Radiographic response, safety | EGFRvIII expression | Phase I – Exploratory (Low evidence) | Feasible; antigen loss and transient responses observed | Completed [85] |
| CAR-T | IL13Rα2 CARv3-TEAM-E (Choi et al., 2024) | Single-arm, intraventricular delivery | Recurrent GBM | 3 | Radiographic/neurologic response | IL13Rα2 expression | Phase I – Exploratory (Low evidence) | Rapid regressions (2 transient, 1 durable response) | Completed [58] |
| OV | G47Δ (Teserpaturev) | Single-arm, open-label | Recurrent/progressive GBM | 19 | 1-yr OS rate | None | Phase II – Preliminary (Approved Japan) | 1-yr OS 92.3%; favorable safety | Approved (Japan) [59] |
| OV | PVSRIPO (poliovirus chimera) | Single-arm, dose-escalation | Recurrent GBM | 61 | OS, safety | None | Phase I – Exploratory (signal of activity) | Durable responses in subset; long-term survivors | Phase II completed [54] |
| ICI + OV | DNX-2401 + Pembrolizumab (CAPTIVE/KEYNOTE-192) | Open-label combination | Recurrent GBM | 49 | Safety, OS | None (unselected GBM) | Phase I/II – Preliminary | Acceptable safety; signal of activity | Ongoing [94] |
| Metabolic Modulation | Epacadostat (IDO inhibitor) | Randomized vs Pembrolizumab (alone) – melanoma reference trial | Advanced solid tumors (preclinical GBM only) | ≈350 (melanoma trial) | OS, PFS | None | Phase III – Negative elsewhere; investigational for GBM | No efficacy in solid tumors; GBM development experimental | Investigational [89] |

Table S1. Expanded dataset corresponding to Table 2. Provides full methodological context including patient population, sample size, endpoints, and biomarker stratification for each cited immunotherapy trial in gliomas. Intended for transparency and to substantiate evidence grading presented in the main text.
